# Identification of inflammatory markers in eosinophilic cells of the immune system: fluorescence, Raman and CARS imaging can recognize markers but differently

**DOI:** 10.1007/s00018-021-04058-4

**Published:** 2021-12-22

**Authors:** Aleksandra Borek-Dorosz, Marek Grosicki, Jakub Dybas, Ewelina Matuszyk, Marko Rodewald, Tobias Meyer-Zedler, Michael Schmitt, Juergen Popp, Kamilla Malek, Malgorzata Baranska

**Affiliations:** 1 Faculty of Chemistry, Jagiellonian University, Gronostajowa 2, 30-387 Krakow, Poland; 2 Jagiellonian Centre for Experimental Therapeutics (JCET), Jagiellonian University, Bobrzynskiego 14, 30-348 Krakow, Poland; 3 Institute of Physical Chemistry (IPC) and Abbe Center of Photonics (ACP), Friedrich-Schiller-University, Helmholtzweg 4, Jena, Germany; 4 Leibniz Institute of Photonic Technology e.V., Member of Leibniz Health Technologies, Albert-Einstein-Str. 9, Jena, Germany

**Keywords:** EoL-1 cells, Primary isolated eosinophils (Eos), Fluorescence microscopy, Spontaneous Raman spectroscopy, Coherent anti-Stokes Raman scattering (CARS)

## Abstract

Eosinophils (Eos) play an important role in the immune system’s response releasing several inflammatory factors and contributing to allergic rhinitis, asthma, or atopic dermatitis. Since Eos have a relatively short lifetime after isolation from blood, usually eosinophilic cell line (EoL-1) is used to study mechanisms of their activation and to test therapies. In particular, EoL-1 cells are examined in terms of signalling pathways of the inflammatory response manifested by the presence of lipid bodies (LBs). Here we examined the differences in response to inflammation modelled by various factors, between isolated human eosinophils and EoL-1 cells, as manifested in the number and chemical composition of LBs. The analysis was performed using fluorescence, Raman, and coherent anti-Stokes Raman scattering (CARS) microscopy, which recognised the inflammatory process in the cells, but it is manifested slightly differently depending on the method used*.* We showed that unstimulated EoL-1 cells, compared to isolated eosinophils, contained more LBs, displayed different nucleus morphology and did not have eosinophilic peroxidase (EPO). In EoL-1 cells stimulated with various proinflammatory agents, including butyric acid (BA), liposaccharide (LPS), or cytokines (IL-1β, TNF-α), an increased production of LBs with a various degree of lipid unsaturation was observed in spontaneous Raman spectra. Furthermore, stimulation of EoL-1 cells resulted in alterations of the LBs morphology. In conclusion, a level of lipid unsaturation and eosinophilic peroxidase as well as LBs distribution among cell population mainly accounted for the biochemistry of eosinophils upon inflammation.

**Supplementary Information:**

The online version contains supplementary material available at 10.1007/s00018-021-04058-4.

## Introduction

Eosinophils (Eos) belong to granulocytes, the type of white blood cells in the innate immune system, characterized by the presence of granules in their cytoplasm. They participate in the regulation of inflammatory conditions by releasing several inflammatory mediators such as cytokines and reactive oxygen species. Eos are also involved in the communication between the immune system cells [1]. They play an important role in allergic rhinitis, asthma, atopic dermatitis, and the immune system’s responses, including fighting parasites. In allergy, Eos are considered to play two opposite roles. They inactivate histamine—a slow reactive substance of anaphylaxis and platelet-activating factor (PAF), which is responsible for attenuation of the allergic inflammation products. But eosinophil peroxidase (EPO) and eosinophil cationic protein present in granules worsen and prolong allergic inflammations by their cytotoxic effects [2]. Mature Eos possess bilobed nuclei [3] and only a few percent of cells have lipid bodies (LBs) in their cytoplasm under normal conditions [3, 4]. LBs are lipid-rich cytoplasmic inclusions, which regulate the storage and hydrolysis of neutral lipids. Rapid formation and accumulation of LBs in cells are also observed in the inflammatory state, including asthma and allergies [5, 6]. LBs play an important role in cell signaling, membrane trafficking, regulation of lipid metabolism, controlling of synthesis and secretion of inflammatory mediators (e.g., eicosanoids) [7]. In Eos, interleukin-5 induces production of LBs that are major intracellular sites for the activation-elicited formation of LTC4 [5]. LBs in inactivated Eos are present on the surface of the ribosomes and ribosome subunit-like particles, and are surrounded by a monolayer of phospholipids with some contribution of proteins [5, 6, 8].

The number of Eos is less than 5% of all leukocytes in the peripheral blood. Their lifespan is short (ca. 18 h). The purification of sufficient numbers of Eos is difficult and time-consuming [9] which impedes in vitro studies on their biological properties [10]. Human eosinophilic leukemia cells (EoL-1) are considered to be an ideal model of eosinophils for in vitro studies [11], in particular for the examination of pathways of their inflammatory responses [12]. However, they exhibit morphological differences compared to Eos (Tab. S1 in SI). Under normal conditions, EoL-1 cells are cytologically similar to myeloblasts without typical bilobed nuclei and low number of granules with EPO [11]. EoL-1 cells are differentiated into mature eosinophil-like cells after treatment with agents such as butyric acid (BA), dibutyryl cyclic adenosine monophosphate (dbcAMP) [10], tumor necrosis factor (TNF-α) [12], interferon-7 (IFN‐7) or phorbol 12-myristate 13-acetate (PMA) [3].

The most often used BA causes nuclear lobulation and an increase of cytoplasm—nucleus ratio and production of granulocytes after 5–7 days (500 μM BA) [10]. [13] Granules contain eosinophil cationic protein as well as some eosinophillotactic attractants such as platelet-activating factor (PAF), interleukin-5 and 3 (IL-5 and IL-3), and Granulocyte Macrophage Colony-Stimulating Factor (GM-CSF).

TNF-α or interleukin 1-β (IL-1β) are also commonly used to differentiate EoL-1 cells. TNF-α activate Eos cells [14] and their migration [15] while Il-1β increases their survival [16]. Both proinflammatory agents induce the release of interleukin-9 and coordinate cellular responses in the asthmatic state observed similarly to Eos from asthmatic patients and their production increases [17]. In turn, stimulation of Eos with interleukin-5 leads to the significant production of LBs [5, 18].

Lipopolysaccharides (LPS) are extracted from gram-negative bacteria and exhibit proinflammatory properties. Some works have reported a LPS effect of on Eos [19–23]. The mechanism of LPS-induced inflammation [24] and inflammation in asthma [25] are similar and might explain mechanism of Eos migration from the periphery to the airways [24] with an increased release of granulocytes [25].

In this work, we applied different microscopic techniques for the analysis of LBs in isolated human Eos and EoL-1 stimulated with various proinflammatory factors important for eosinophilic action in the body. EoL-1 cells were used as a model of human Eos because there are few Eos cells in the blood and their lifespan is short. To evoke the production of LBs, BA, IL-1β, LPS, and TNF-α were used. First, we examined the cells with 2D fluorescence microscopy as conventionally done by biologists and pharmacologists and then we employed two imaging techniques, coherent anti-Stokes Raman effect (CARS) and spontaneous Raman scattering (RS) to quantify LBs and determine their composition. This multimodal approach allowed assessing for the first time morphological and compositional features of stimulated Eol-1 in comparison to mature Eos. We also discussed benefits of each technique for further studies of biochemical features in granulocytes.

## Materials and methods

### Chemicals

Dextran from Leuconostoc spp. 500 000 (Sigma-Aldrich), Ficoll-Paque Plus *d* = 1.077 g/ml (GE Healthcare,), human eosinophil isolation kit (MiltenyiBiotec), DPBS (Dulbecco’s phosphate-buffered saline, Gibco), 10% fetal bovine serum (FBS, Gibco), penicillin–streptomycin (10 000 U/mL, Thermo-Fisher Scientific), Roswell Park Memorial Institute (RPMI) 1640 medium with L-glutamine and 25 mmol/l HEPES (Gibco), EDTA (Sigma-Aldrich), butyric acid (BA, Sigma-Aldrich); TNF-α (Tumour Necrosis Factor, Sigma-Aldrich), Il-1β (Interleukin 1β, Sigma-Aldrich) and LPSs (lipopolysaccharides, Sigma-Aldrich).

### Cell culturing

Human Eos were isolated according to the method described by Grosicki [26]. Briefly, human blood was purchased from the Regional Blood Transfusion Center and stored in heparin-coated tubes. Erythrocytes (RBCs) were discarded from the sample, through 1% dextran solution sedimentation [dextran from Leuconostoc spp. 500,000 (Sigma-Aldrich)]*.* PBMCs were removed by Ficoll-Paque density gradient separation (Ficoll-Paque Plus *d* = 1.077 g/ml (GE Healthcare). The remaining RBCs were eliminated through hypotonic shock lysis. Eos were further purified through the immunomagnetic cell separation method (human eosinophil isolation kit MultiantiBiotec). The immunomagnetic separation was performed according to the manufacturer's instruction. Cells after isolation were counted and used immediately for fluorescence microscopy. For other experiments, cells were fixed in 2.5% glutaraldehyde dissolved in PBS.

EoL-1 cells (ATCC, Manassas, VA, USA) were grown in RPMI 1640 cell culture medium, supplemented with 10% (v/v) FBS, 2 mmol/l l-glutamine and penicillin–streptomycin (10 000 u/ml). Cells were grown for 7 days and passaged when the cellular density reached 2.0 × 10^6^ cells/ml. During the passage, cells were diluted to 0.5 × 10^6^ cells/ml. Subsequently, EoL-1 cells were treated either with 50 μM BA for seven days (Fig. S1, Supplementary Information), and with 10 ng/ml TNF-α, Il-1β, and LPS for 24 h. All cells were incubated at 37 °C and 5% CO_2_. After the incubation time cells were fixed with 2.5% glutaraldehyde solution for 10 min and suspended in PBS.

### Raman spectroscopic imaging

Raman imaging was carried out with the use of a WITec confocal Raman imaging system (WITec alpha 300 Raman microscope). Raman spectra were acquired with an excitation laser at 532 nm, which was coupled to the microscope via an optical fiber with a core diameter of 50 µm. The microscope was equipped with a CCD detector cooled to – 80^°^ C. Cells immersed in PBS solution and placed on a CaF_2_ window were illuminated through a 60 × water objective (NA: 1.0). Raman images were recorded with a step size of 0.5 μm and laser power of 23 mW. Raman spectra were collected with an integration time of 0.5 s and a spectral resolution of 3 cm^−1^. For each experimental group images of 15 cells were analyzed. But to examine intragroup variability we investigated three independent replicates for control and BA groups, and one replicate for TNF, LPS and IL-1β. Typical Raman spectra of cytoplasm in the control and BA groups were displayed in Fig. S2 (Supplementary Information) and they confirm good reproducibility of spectral features between replicates. One of these replicates was tested with all three methods sequentially, i.e. fluorescence, Raman and CARS imaging, to show they are truly complementary, and these data are shown in the manuscript. Raman spectra were preprocessed using a cosmic ray removal filter (filter size 3 and dynamic factor 8) and baseline correction (polynomial, grade 3). Afterwards, k-means cluster analysis (KMC) was performed using a WITec 5.0 software.

### Fluorescence microscopy

Fluorescence measurements were performed on an Olympus ScanR automated fluorescence microscope (20×, NA = 0.5). LBs were stained with BODIPY 493/503 (ThermoFisher) (excitation 500–650 nm, emission 510–665 nm), and nuclei with Hoechst 33,342 (ThermoFisher) (excitation 360 nm, emission 497 nm). Cells were seeded on 96-well plates, coated with a poly-l-lysine solution and centrifuged (300 × *g*, 5 min at room temperature) to deposit cells on the bottom of the plate. Before measurements, cells were fixed with 4% paraformaldehyde (10 min.), washed gently with DPBS, and stained with 4 µg/mL BODIPY 493/503 dye for 30 min. at room temperature. Then, cell nuclei were stained with Hoechst 33,342 (0.5:1000) and then cells were imaged using a fluorescence microscope. Experimental data were collected from four independent biological experiments. Images were analyzed using a Columbus 2.4.2 image data storage and analysis system software (PerkinElmer). The number of LBs were determined based on the BODIPY signal using an option ‘Find spots’ (method B, area > 2 µm^2^) in a Columbus software. The number of cells were calculated based on the number of nuclei visualized by the Hoechst staining (option ‘Find nuclei’ in the Columbus software, method B, area > 5 µm^2^). All respective graphs plotted to show morphological features of LBs and nuclei were interpreted as mean values with standard deviations calculated for four fluorescence images.

### Coherent anti-Stokes Raman scattering and two-photon excited fluorescence microscopy

The experimental platform for CARS imaging has been described in detail previously [27]. To obtain an image, the symmetric CH_2_ stretching vibration at 2850 cm^−1^ was chosen. The pump beam was tuned to 672.5(7) nm (42 mW @sample), and the Stokes was 832(2) nm (32 mW @sample). For recombination of these two beams with temporal and spatial overlap a delay stage was used in combination with a retro reflector, and an 800 nm long pass dichroic mirror. The beams were directed into the laser scanning microscope (LSM 510 Meta, Zeiss, Germany). Samples were illuminated using a 40 × water immersion objective (1.1 NA, LD-C-Apochromat, Zeiss, Germany). The CARS signal in this experiment was collected in forward direction by an NA 0.8 condenser, filtered by a bandpass filter (550(88) nm), and detected by a photomultiplier (Hamamatsu R6257, Japan). Alongside the CARS signal, two-photon excited fluorescence (TPEF) was collected in epi direction by the focusing objective, separated from the excitation beam by a beam splitter and filtered by a band pass filter (458(64) nm). The signal was detected using a photomultiplier (Hamamatsu R6257, Japan) using the same integration time and number of pixels as for the CARS channel. The field of view was 112.5 µm × 112.5 µm, sampled with a resolution of 1024 × 1024 pixels at an integration time of 1.6 µs per pixel. For every image, 16 frames were averaged. For each cell group at different positions of the sample (13 for control, 15 for BA, 18 for TNF, 16 for IL-1β and 19 for LPS). One replicate of cell culturing was investigated here because Raman and fluorescence imaging indicated low intragroup variability. Two-photon excited fluorescence (TPEF) images (458 bp 64 nm) were recorded simultaneously.

### Identification of lipid bodies in CARS images

Lipid bodies were identified using the FIJI (1.52v) distribution of ImageJ [28, 29] and a revised version of a previously detailed [30] custom ImageJ macro. Briefly, the images were subjected to background subtraction using FIJIs built-in rolling ball method and a ball radius of 3 px. The Laplacian of the image was calculated using the FeatureJ plugin [31] and a smoothing scale of 1. The Laplacian image was subjected to an FFT bandpass filter as implemented in ImageJ with the lower band edge corresponding to 2 px and the upper band edge to 20 px. The resulting image was inverted and converted into 8-bit format. Depending on the set of cells, global thresholding was applied using either FIJIs built-in ‘Auto-Threshold’ function and the ‘RenyiEntropy’ method with default parameters (control, LPS, TNF), or 1.4 times the average brightness of the image as threshold (BA50, IL-1beta), where more fine control was necessary. The obtained binary images were analyzed using ImageJs ‘Analyze Particles…’ function and any areas with either a circularity of less than 0.5 or an area of less than (2 px)^2^×π/4 or more than (20 px)^2^×π/4 were excluded. The final number of LBs was estimated to be the number of remaining separated bright areas. The script is part of Supporting Information and contains a detailed documentation.

### Cell identification in CARS/TPEF images and per-cell analysis of LB counts

To obtain per-cell statistics of the number of LBs, cells were identified in a semi-automated multi step procedure. To reduce the amount of manual labor, composite RGB images generated from the two-channel CARS/TPEF images were subjected to classification by the trainable WEKA segmentation algorithm (v 3.2.34, [32]) as implemented in the FIJI distribution of ImageJ using all available features and otherwise default parameters. RGB images (red: CARS, green: TPEF) were generated after rescaling the individual channels using the ImageJ’s “Enhance Contrast” function, allowing for 3.5% saturation, before down converting the 16-bit images to 8 bit. The WEKA algorithm was trained on two manually classified images using two classes (background and foreground). The resulting cell masks were cleaned in a two-step procedure: first, in an automated step, all holes smaller than 500 px in congruent areas were filled, a watershed segmentation was performed and all congruent areas smaller 2500 px, or ones touching the image edges were excluded. In the second step, the obtained masks were overlaid with the RGB images and manually corrected. All areas belonging to cell fragments, dead cells or cell clusters with unclear boundaries/strong overlap were excluded and instances of inaccurate assignment or suboptimal segmentation during the watershed segmentation were rectified. An overview of the above-described procedure was provided in Fig. S3. Following cell identification, the number of LBs, identified as detailed above, per cell was calculated.

## Results

### Primary eosinophils (Eos) versus eosinophilic cell line (EoL-1)

**Fig. 1:**
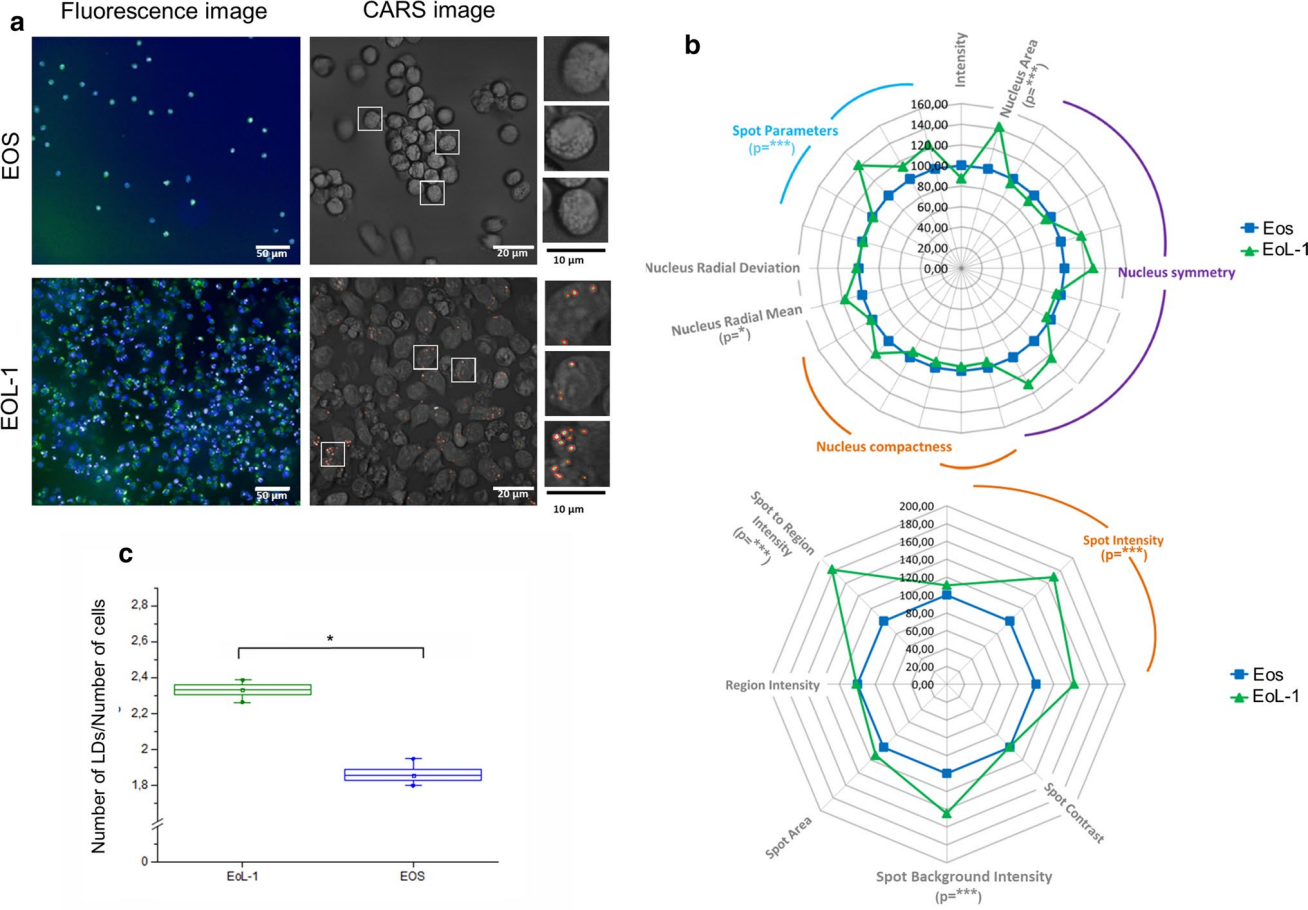
**a** Fluorescence (Hoechst nuclei—blue and BODIPY LBs—green, magnification 20×, NA = 0.5) and coherent anti-Stokes Raman scattering images (CARS, magnification 40×, NA = 0.8) of primary Eos and unstimulated EoL-1. **b** Multi-parametric morphological analysis of nuclei (upper) and LBs (lower) from fluorescence images. The significance was calculated with the Mann–Whitney test (*p < 0.05, **p < 0.01, ***p < 0.001) and it is shown underneath the parameters in brackets. **c** The relative number of LBs, shown as a graph of the number of LBs per number of cells, calculated from fluorescence images (N of EoL-1: 1430, N of Eos: 157). Values are given as mean ± SEM (standard error of mean) and are shown in box plots: mean (horizontal line), SEM (box), minimal and maximal values (whiskers). The significance was calculated with the Mann–Whitney test (*p < 0.05)

In our previous reports, we observed by spectroscopic imaging that native human Eos isolated from the peripheral blood contain a sparse population of LBs, which is congruent with their morphology and function [3, 4]. Here, we determined for the first time the distribution and number of LBs in Eos and unstimulated EoL-1 cells with the use of lipid-sensitive CARS and fluorescence imaging (Fig. 1a and c). According to the analysis of BODIPY-stained cells, the average number of LBs per cell was 2.35 ± 0.05 and 1.85 ± 0.06 (mean ± SEM) for EoL-1 and Eos, respectively. The quantification of LBs from CARS images showed similar results but also revealed large cell-to-cell variances (data not shown). An unsaturation level of lipids accumulated in LBs is slightly higher in isolated Eos than EoL-1 (Fig. S4 in SI). The determined number of the C = C bonds in the acyl fatty acid chain was 1.5. Unsaturation of lipids was estimated based on spectra of LBs collected with spontaneous Raman microscopy. Morphological differences, revealed by fluorescence staining, between Eos and EoL-1 cells were additionally determined for LBs and nuclei using versatile Columbus 2.4.2 morphological image analytical algorithms. In that way, we assessed parameters of these cellular compartments like symmetry, intensity, spot intensity, and area, etc., both, for Eos and Eol-1 cells. EoL-1 fluorescence features were used as a reference (Fig. 1b). The Eos nuclei were smaller, more compact, and less symmetrical than in EoL-1 cells. The fluorescence signal of the BODIPY dye in LBs was brighter and covered a larger area in the EoL-1 cell line than in Eos isolated from blood. In the literature data about the differences in those cells concerning size and abundance of LBs in both, Eos and EoL-1, are in agreement with our findings [3, 8, 18, 32].

### Lipid bodies in EoL-1 cells stimulated by a proinflammatory factors

**Fig. 2:**
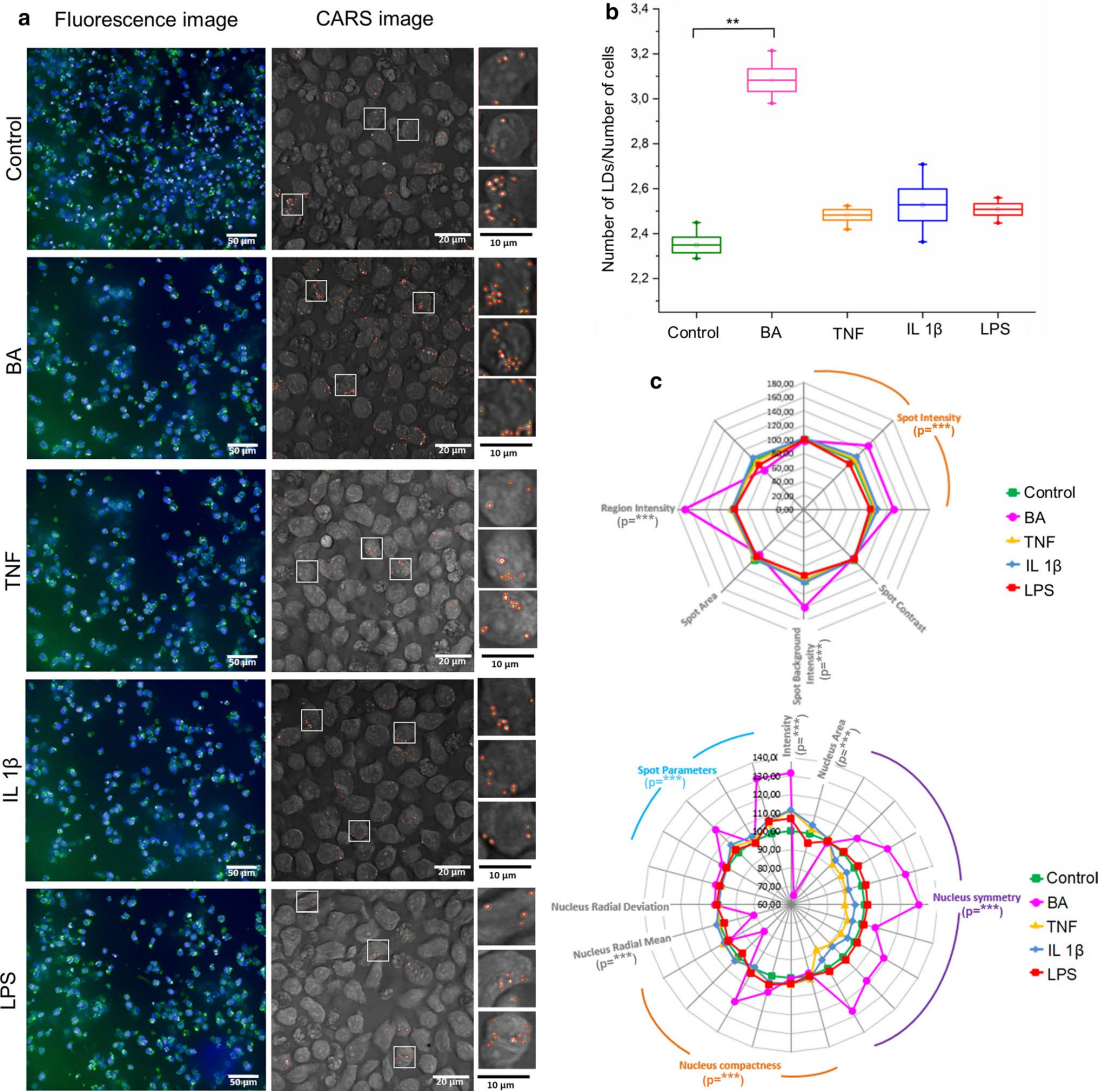
**a** Fluorescence (Hoechst nuclei—blue and BODIPY LBs—green, magnification 20×, NA = 0.5) and coherent anti-Stokes Raman scattering images (CARS, magnification 40×, NA = 1.1) for control and stimulated EoL-1 cells. **b** The relative number of LBs, shown as a graph of the number of LBs per number of cells, calculated from fluorescence images (N of control cells: 1430, BA: 2620, TNF: 870, Il-1β: 840, LPS: 3730). Values are given as mean of the 4 images means ± SEM between the images and are shown in box plots: mean (horizontal line), SEM (box), minimal and maximal values (whiskers). *p < 0.05, **p < 0.01, ***p < 0.001. **c** Multi-parametric morphological analysis of LBs (upper) and nuclei (lower) from fluorescence images. The significance (*p < 0.05, **p < 0.01, ***p < 0.001) refers only to comparison of control and BA cells and it is shown underneath the parameters in brackets

Fluorescence microscopy and CARS imaging were first employed to visualize LBs formed due to stimuli of proinflammatory action, i.e. BA, TNF-α, IL-1β, and LPS. Data from fluorescence imaging were recorded from four independent biological experiments, while 13–19 images from different positions in one cell culture were recorded by CARS (Fig. 2a). The fluorescence intensity of the BODIPY dye counted from ca. 2000–3000 cells confirmed the CARS results. The average number of LBs per cell increased significantly to 3.08 ± 0.1 due to incubation with BA compared to 2.3 ± 0.07 in non-treated cells, see Fig. 2b. Stimulation of EoL-1 cells with IL-1β, TNF-α, and LPS resulted in the formation of only few new LBs; the average number of LBs/cell in those cells was 2.4–2.5. Our findings for BA-stimulated cells agreed with several reports showing that the formation of LBs could be an indicator of the EoL-1 differentiation into Eos [10, 13, 33, 34] due to the alteration of lipid metabolism, the composition of fatty acid, and the autophagy process [35–37]. Next, the multi-parameter analysis of fluorescence images was performed for both, nucleus and LBs in respect to control EoL-1 cells (Fig. 2c). The biggest changes were evoked by the BA stimulation that caused a pronounced distortion of nucleus symmetry whilst IL-1β and TNF-α induced weaker transformations of the nuclei’s shape and size. LPS-treated EoL-1 nuclei were similar to control. The corresponding fluorescence analysis of LBs signal showed that their size and irregular shape, probably resulting from the aggregation of LBs, were enormously enlarged in BA-treated cells (Average ± SEM, control: 2.35 ± 0.03, BA: 3.11 ± 0.05, TNF: 2.43 ± 0.02, IL 1β: 2.49 ± 0.07, LPS: 2.44 ± 0.02).

### Analysis of LBs in stimulated EoL-1 from CARS images

**Fig. 3:**
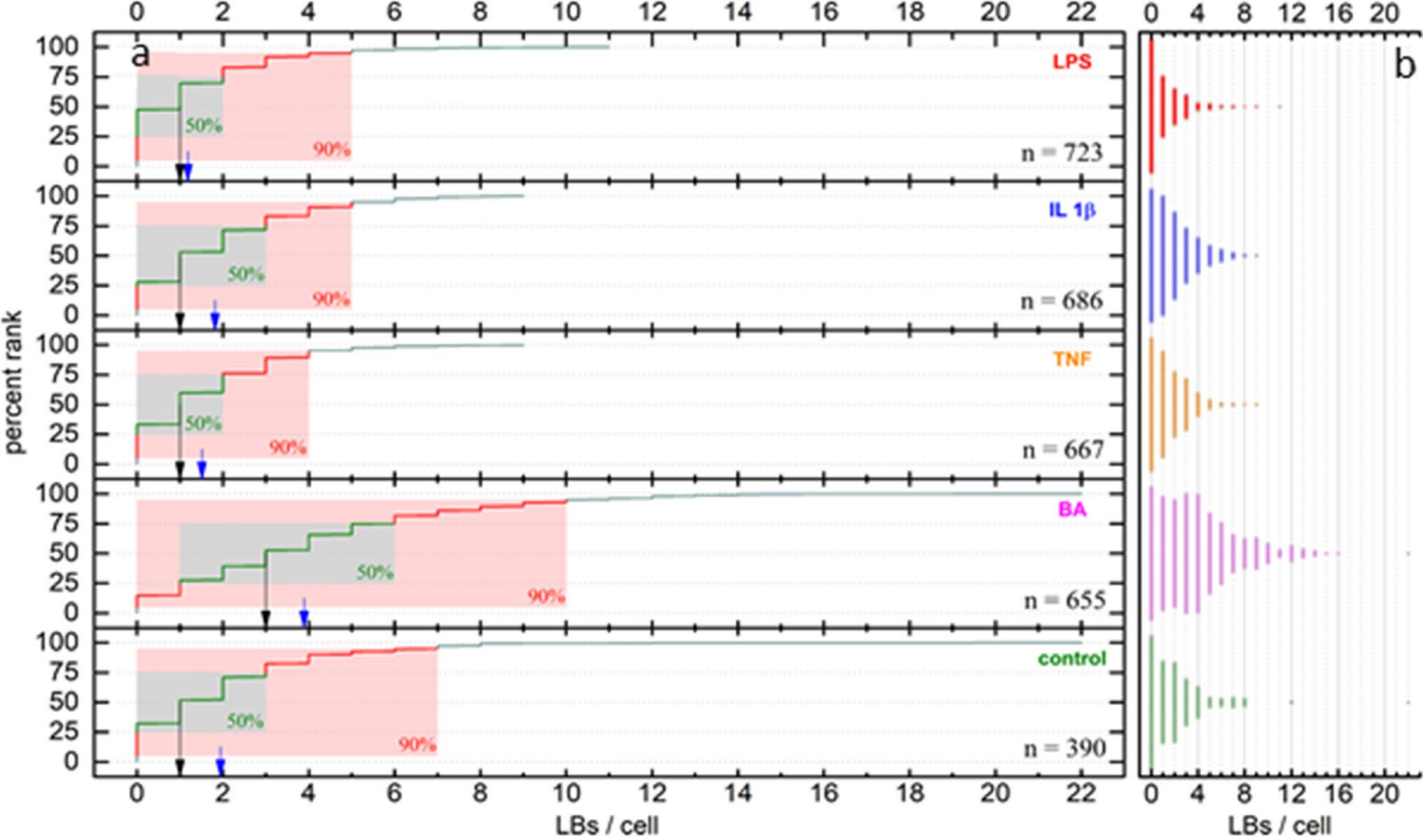
**a** Percent rank analysis of LBs per cell in stimulated EoL-1 cells as determined by CARS microscopy. Black arrows indicate median values, blue arrows indicate mean values. Green boxes cover the 25–75 percentile range, red boxes the 5–95 percentile range, n refers to the number of measured cells. **b** Distribution of LB counts per cell

While the analysis of fluorescence images provided a holistic view, i.e., an average number of LBs per cell over all cells in each image, it did not provide information about the cell-to-cell variation. For that reason, CARS images were analysed on a per-cell basis (Fig. 3, Table S2 in SI). We found that for all stimulation methods and the control sample the largest fraction of cells did not have any LBs in the focal plane while the remaining cells showed varying numbers of LBs (typically 1–8) with exceptional cases showing up to 22 LBs in a single cell. This strong deviation from a normal distribution does not allow for a characterisation of the data dispersion in terms of standard deviations. A characterization based on simple percentiles proved similarly limited in its ability to provide visual intuition of the various distributions as for all treatment groups except BA the first percentiles *P* for all *P* < 25% fall together, with the LPS treatment group even including all *P* < 47%.

Thus, a percent rank analysis was performed as depicted in Fig. 3a, providing an easy to grasp visual representation of the dispersions involved. Median [1 for all treatment groups, except BA (3)] and mean (control: 1.94, BA: 3.89, TNF: 1.51, IL 1β: 1.82, LPS: 1.18) LBs/cell numbers are reported as location parameters and depicted in Fig. 3a as black and blue arrows, respectively. As for the fluorescence microscopy analysis, these data suggest a strong effect on LBs production upon stimulation with BA, whereas the remaining stimulation groups are similar to the control group in this regard. While these data are in qualitative agreement with those obtained from fluorescence microscopy, it is important to note that quantitative comparability between the results obtained from the fluorescence microscopy images with those based on CARS/TPEF microscopy is limited due to the differences in technique, analysis approach and instrumentation. Given that neither of the methods guaranties that all LBs present in a given cell are detected, either because they are not located close enough to the focal plane, or because the (semi-) automated analysis procedures did not correctly identify them, it appears likely that both methods underestimate the true number of LBs/cell. This, however, does not limit comparability within the respective techniques.

### Chemical features of intracellular compartments of stimulated EoL-1 cells

**Fig. 4:**
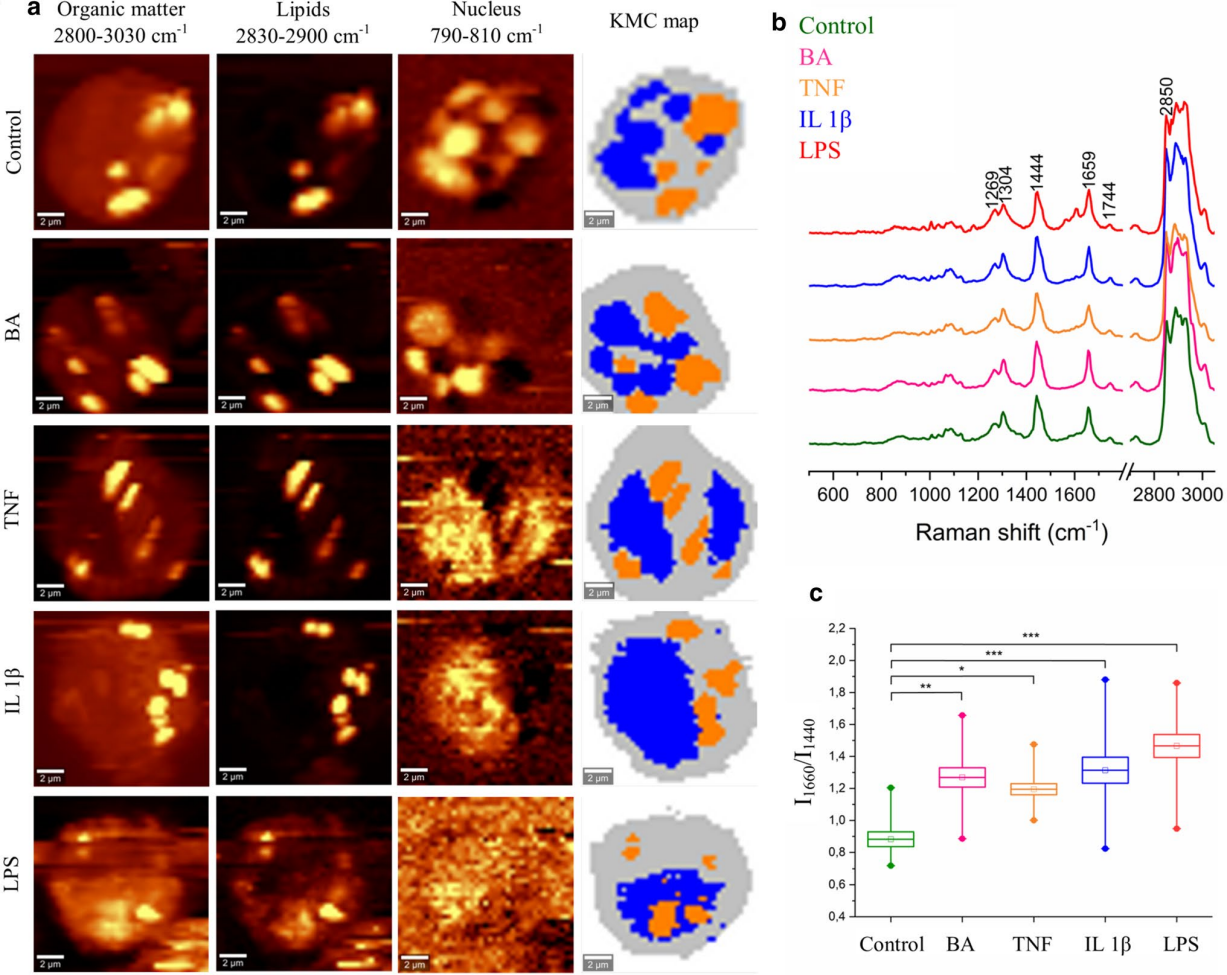
**a** Raman distribution images of selected chemical components: organic matter, lipids, and DNA and false-colour KMC map of representative cells of control and stimulated EoL-1 cells. Three classes were assigned to the cytoplasm (grey), LBs (orange), and nuclei (blue). Raman distribution images were constructed by integration of characteristic bands for organic matter (2800–3030 cm^-1^), lipids (2830–2900 cm^-1^), and nuclei (780–800 cm^-1^). **b** Mean spectra (*n* = 15) ± SD (shading) of the LB class for each group of cells, with assigned Raman bands characteristic for lipids, (**c**) analysis of the degree of unsaturation of the lipid class in EoL-1 cells calculated using the integrated intensity ratio of bands 1660 and 1440 cm^-1^. Values are given as mean ± SEM and are shown in box plots: mean (horizontal line), SEM (box), minimal and maximal values (whiskers). *p < 0.05, **p < 0.01, ***p < 0.001

Fluorescence microscopy and CARS imaging are techniques that rely on the detection of specific cellular components, like DNA in the nucleus, and lipids in the cytoplasm, whereas label-free spontaneous Raman imaging provides a simultaneous response from all biomolecules in the focal volume. This method combined with uni- and multivariate analysis revealed the chemical composition of stimulated and control EoL-1 cells (Fig. 4). Raman images were constructed by integration of the CH/CH_2_/CH_3_ stretching modes in the high wavenumber region, which is specific for all cellular biomolecules. To visualize LBs, Raman images were constructed by band integration of the CH_2_ stretching modes while bands assigned to the breathing vibrations of T, C nucleobases and the O–P–O backbone mode were chosen to show the nucleus distribution (Fig. 4a). Non-hierarchical k-means cluster analysis (KMC) divided the spectra of the whole cell into clusters of nuclei, LBs, and cytoplasm (Fig. 4a). Figure 4b displays averaged Raman spectra of the LB classes extracted from KMC analysis for all groups of cells. These spectra show typical Raman features of the acyl chains in lipids [38], i.e. bands at 2850 and 3030 cm^−1^ attributed to the stretching vibrations of the methylene [ν(C–H)] and olefinic moieties [ν(= C–H)], respectively, and vibrations in the fingerprint region at positions of 1660 cm^−1^ [ν(C = C)], 1440 cm^−1^ ([scissoring mode: δ(CH_2_/CH_3_)], 1304 cm^−1^ [twisting mode: τ(CH_2_)], and 1269 cm^−1^ [deformation vibration: β(= CH)] [38]. In addition, bands of triacylglycerols [1744 cm^−1^, ν(C = O)] and low intensity signals from cholesterol esters [974 cm^−1^, β(CH)] were observed. The ratio of band intensities centered at 1660 and 1440 cm^−1^ served to estimate unsaturation level of lipids [38]. An ANOVA analysis indicated the production of unsaturated fatty acids due to the action of all proinflammatory agents (Fig. 4c).

**Fig. 5:**
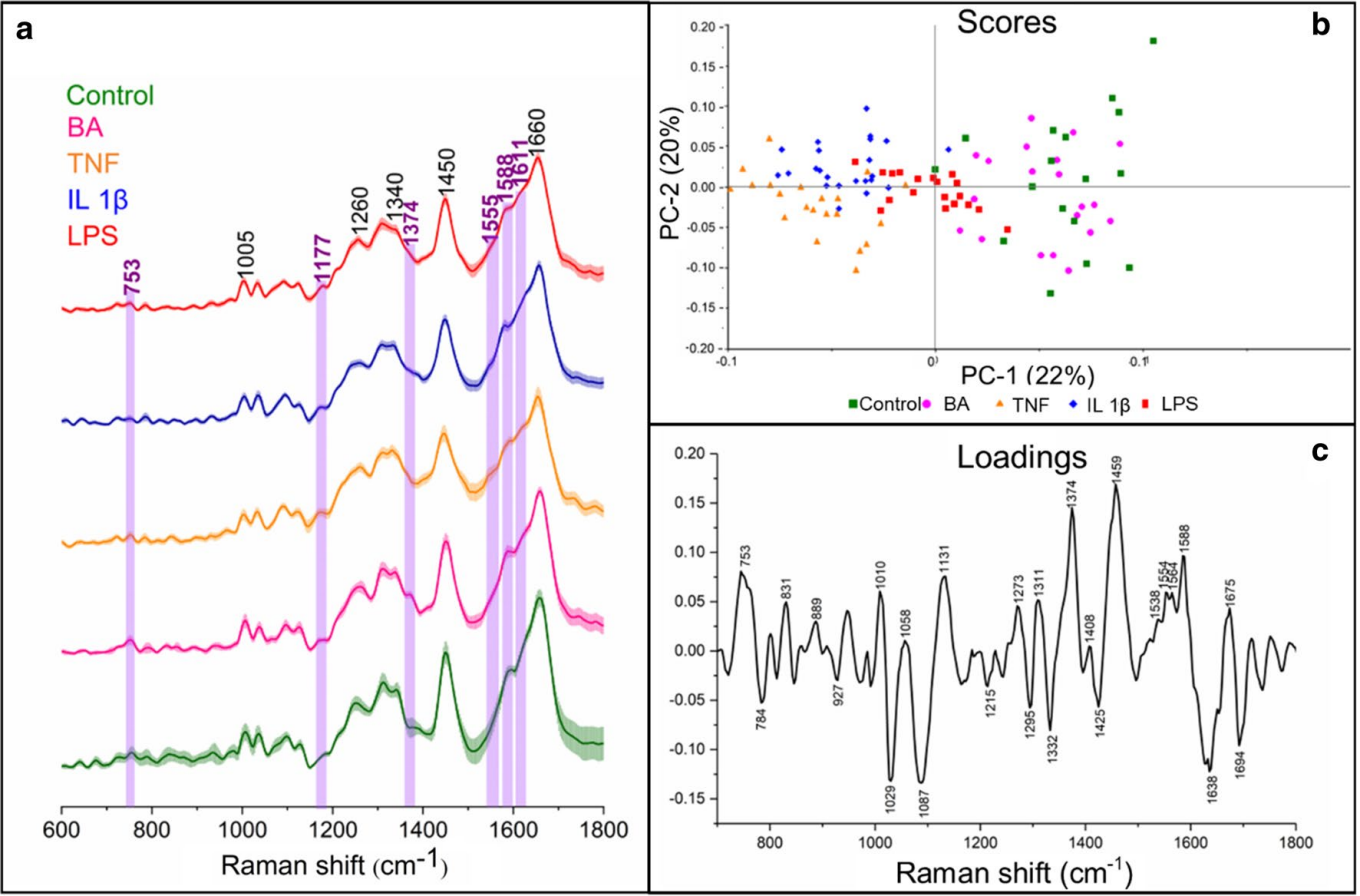
**a** Mean spectra (*n* = 15) of the cytoplasm class for each group of cells with standard deviation (shading); bands assigned to EPO are marked in violet. **b** Scores and (**c**) loading plots of principal component analysis performed in the region of 600–1800 cm^-1^

Next, we examined biochemical changes in the nucleus and cytoplasm regions (Fig. 4a, KMC, blue and grey classes, respectively). The Raman features of nuclei did not show any chemical differences (Fig. S5 in SI), contrary to cytoplasm (Fig. 5a). For the latter, subtle variation of band intensities among the experimental groups was observed and we noted the presence of resonance Raman signals of eosinophil peroxidase (EPO)—the heme protein specific for Eos (marked in violet). The most pronounced EPO bands were observed in cytoplasm of EoL-1 cells incubated with LPS. Since the spectral differences in the intensity of Raman bands were subtle, we employed principal component analysis (PCA) (Fig. 5b and c). A score plot for PC-1 and PC-2 showed the segregation of the cells along PC-1 with a variation of 22% (Fig. 5b). Control and BA-stimulated EoL-1 cells were grouped along PC-1(+) whereas cells treated with TNF and IL-1β on the opposite site along PC-1(−). LPS-incubated cells were centered and separated from other groups. A PC-1 loading plot showed the main spectral discriminators of the differentiated groups (Fig. 5c). Control and BA-treated cells were distinguished due to Raman bands at 753, 1311, 1374, 1554, 1564, and 1588 cm^−1^ assigned to eosinophil peroxidase [4] and a high-intensity band at 1459 cm^−1^ (proteins) [39]. The bands at 1367 and 1588 cm^−1^ suggested that the presented heme group in those cells possesses a 6-coordinated ferric heme group, which is characteristic for EPO [4]. The group of cells clustered on the PC-1(-) axis of the loadings plot was differentiated from the others due to features of nucleic acids, lipids, and proteins, i.e. bands at 784 cm^−1^ (cytosine, uracil, thymine), 1029 cm^−1^ (proteins crosslinking) [40], 1087 cm^−1^ (phospholipids) [41], 1332 cm^−1^ (CH_2_ moieties in proteins, lipids, and nucleic acids), 1425 cm^−1^ (CH_3_ moieties in proteins and lipids) [40], 1638 cm^−1^ (oxygenated cells) [42], and 1694 cm^−1^ (amide I) [43].

## Discussion

Eosinophils in peripheral blood constitute around 5% of all leukocytes and their lifespan is very short (approx. 18 h) which impedes studies of their biological properties [9]. The EoL-1 cell line is a commonly used model to study Eos responses under different conditions [11, 12]. The application of fluorescence, CARS, and Raman microscopy allowed us to examine the eosinophils’ most important components including nuclei, LBs, and EPO that appeared due to the stimulation with proinflammatory agents. LBs play a key role in the functioning and activity of Eos. Fluorescence microscopy indicated that even unstimulated EoL-1 possesses more LBs than primary eosinophils (Fig. 1a and b). Moreover, the in vitro model of Eos differed from the cells present in the human body by nucleus symmetry and compactness as well as spot parameters of LBs. As was mentioned before, also morphologically the cells differed, especially with respect to the nuclei (Fig. 1c).

The morphological and biochemical properties of unstimulated EoL-1 cells changed due to stimulation with four proinflammatory agents acting on cells through different mechanisms. The multi-parametric morphological examination of EoL-1 cells revealed that only BA affected nucleus compactness and symmetry as well as size and shape of LBs (Fig. 2c). Interestingly, EoL-1 cells responded to stimulation with BA by the production of numerous LBs in contrast to IL-1β, TNF-α, and LPS. This suggested a strong inflammation as observed previously in various cell cultures, in particular in the endothelium [4, 5, 18, 44]. Considering the use of the EoL-1 cell line in studies involving the function of LBs our results suggest that any stimulation of the model cells differs in this respect from the primary eosinophils.

Even though Raman spectroscopic imaging is a relatively time-consuming method, it delivered important information about the biochemical composition of cellular compartments in EoL-1 cells resulting from stimulation with the respective agents. Our previous report showed that Raman spectra revealed the presence of LBs and EPO in matured EoL-1 cells as in eosinophils [3]. The content of EPO, however, was lower in the EoL-1 cell line than in Eos. Here, we found that chemism of EoL-1 nuclei did not change due to the action of proinflammatory factors (Fig. S5) whereas EPO was identified in the cytoplasm of all experimental groups at higher levels than in control cells and those treated with butyric acid (Fig. 5). This result suggested that the inflammation-induced IL-1β, TNF-α, and LPS affect the content of this heme protein specific for eosinophils.

Raman spectra of LBs showed that they contained fatty acids and triacylglycerols (Fig. 4). No Raman features of phospholipids or cholesterol and its esters were found. Regardless of the action mechanism of the proinflammatory agents, they caused the synthesis of unsaturated lipids [10, 15, 16]. The biggest differences compared to the control cells were evoked by LPS and IL-1β (***p < 0.001). Cells stimulated with BA had also changed the unsaturation ratio (**p < 0.01), while the smallest impact on changes of lipid unsaturation was observed in the cells treated with TNF-α (*p < 0.05). The degree of unsaturation was determined based on intensities of bands specific for the C = C double bonds (1660 cm^−1^) and the methylene groups (1440 cm^−1^) in the acyl chain of fatty acids and it increased to a similar level due to the action of all proinflammatory agents. With the help of the relationship between the 1660/1440 cm−1 ratio and the number of the C = C bonds in known unsaturated fatty acids (Fig. S4), we found that LBs of control cells possess fatty acids with an average number of the C = C bands between oleic (18:1) and linoleic acid (18:2) [18, 45]. LPS, BA, and IL-1β induced the production of a lipid fraction with an unsaturation degree between linoleic and α-linolenic acid, while LBs in the TNF-α-treated cells exhibited properties similar to linoleic acid. The changes in the unsaturation are shown as mean values for examined cells and for references (fatty acids) (Fig. S4).

## Conclusions

Herein, we report, a multi-method approach of spontaneous Raman, CARS, and fluorescence microscopies used to assess biochemical features of primary isolated eosinophils (Eos) and EoL-1 cells, a model cell line of eosinophils. First, we show that the three methods provide information about the cellular response to inflammation, but it is manifested slightly differently depending on the method used. Namely, conventional fluorescence microscopy assesses the morphological characteristic of stained nuclei and LBs in these blood cells and estimates an averaged number of LBs present in control and induced by proinflammatory agents. In turn, label-free CARS imaging with similar time of data collection as for fluorescence microscopy, indicates the detailed distribution of lipid bodies within cells and its results can be semi-automatically used for the quantification of LBs in each cell individually. This approach shows a significant variation in the LBs production upon particular inflammation stress. In line with this finding, fluorescence and CARS imaging revealed that one of the major differences between Eos and EoL-1 was the higher number of accumulated LBs in the latter, which should be highly considered before the use of EoL-1 as a model system. In contrast, additionally, spontaneous Raman shows the difference in the presence of EPO and LBs unsaturation. The activation of EoL-1 with proinflammatory stimuli, i.e., BA, IL-1β, TNF-α, and LPS resulted in a further increase in the number of LBs, particularly after treatment with BA. However, the degree of unsaturation of fatty acids slightly differed between the different stimulated groups of cells as indicated by spontaneous Raman spectroscopy. Undoubtedly, further analysis, for example by mass spectrometry and high-performance liquid chromatography, would be needed to determine the composition of the lipid fractions. Examination of the cytoplasm with the use of chemometric methods (PCA) exhibited subtle changes, mostly due to the presence of EPO. As we showed, an assessment of biological changes in inflamed cells is possible with the use of an innovative approach based on Raman spectroscopy (spontaneous and CARS) and fluorescence microscopy since all of them provided specific information complemented each other.

## Supplementary Information

Below is the link to the electronic supplementary material.

Supplementary file1 (DOCX 1373 KB)

## Data Availability

The datasets generated during and/or analysed during the current study are available from the corresponding author on reasonable request.

## References

[1] Choi Y, Jeon H, Yang EA (2019). Nasal eosinophilia and eosinophil peroxidase in children and adolescents with rhinitis. Clin Exp Pediatr.

[2] Morita M, Saito H, Honjo T (1991). Differentiation of a human eosinophilic leukemia cell line (EoL-1) by a human T-cell leukemia cell line (HIL-3)-derived factor. Blood.

[3] Rygula A, Fernandes RF, Grosicki M (2019). Raman imaging highlights biochemical heterogeneity of human eosinophils versus human eosinophilic leukaemia cell line. Br J Haematol.

[4] Dorosz A, Grosicki M, Dybas J (2020). Eosinophils and neutrophils-molecular differences revealed by spontaneous Raman. CARS Fluoresc Microsc Cells.

[5] Bozza PT, Viola JPB (2010). Lipid droplets in inflammation and cancer. Prostaglandins Leukot Essent Fat Acids.

[6] Weller PF (2016). Leukocyte lipid bodies—structure and function as “Eicosasomes”. Trans Am Clin Climatol Assoc.

[7] Wan H-C, Melo RCN, Jin Z (2007). Roles and origins of leukocyte lipid bodies: proteomic and ultrastructural studies. FASEB J.

[8] Melo RCN, Weller PF (2014). Unraveling the complexity of lipid body organelles in human eosinophils. J Leukoc Biol.

[9] Ip WK, Wong CK, Lam CWK (2003). Tumour necrosis factor-α-induced expression of intercellular adhesion molecule-1 on human eosinophilic leukaemia EoL-1 cells is mediated by the activation of nuclear factor-κB pathway. Clin Exp Allergy.

[10] Jung Y (2015). Comparative analysis of dibutyric cAMP and butyric acid on the differentiation of human eosinophilic leukemia EoL-1 cells. Immune Netw.

[11] Mayumi M (1992). EoL-1, a human eosinophilic cell line. Leuk Lymphoma.

[12] Goldstein LA, Evanoff HL, Kunkel SL (1996). TNF-induced IL-8 and MCP-1 production in the eosinophilic cell line, EOL-1. Mediators Inflamm.

[13] Saito H, Hayakawa T, Mita H (1993). Effect of butyric acid on induction of differentiation into eosinophil-like cells in human eosinophilic leukemia cells, EoL-1 cell line: possible role of granulocyte-macrophage colony-stimulating factor as an autocrine differentiating factor. Int Arch Allergy Immunol.

[14] Saetta M, Di Stefano A, Maestrelli P (1994). Airway eosinophilia in chronic bronchitis during exacerbations. Am J Respir Crit Care Med.

[15] Lampinen M, Carlson M, Sangfelt P (2001). IL-5 and TNF-α participate in recruitment of eosinophils to intestinal mucosa in ulcerative colitis. Dig Dis Sci.

[16] Hallsworth MP, Soh CPC, Twort CHC (1998). Cultured human airway smooth muscle cells stimulated by interleukin-1 β enhance eosinophil survival. Am J Respir Cell Mol Biol.

[17] Gounni AS, Nutku E, Koussih L (2000). IL-9 expression by human eosinophils: regulation by IL-1β and TNF-α. J Allergy Clin Immunol.

[18] Melo RCN, Weller PF (2016). Lipid droplets in leukocytes: organelles linked to inflammatory responses. Exp Cell Res.

[19] Anastase-Ravion S, Blondin C, Cholley B (2003). Heparin inhibits lipopolysaccharide (LPS) binding to leukocytes and LPS-induced cytokine production. J Biomed Mater Res Part A.

[20] Penido C, Castro-Faria-Neto HC, Vieira-de-Abreu A (2001). LPS Induces eosinophil migration via CCR3 signaling through a mechanism independent of RANTES and eotaxin. Am J Respir Cell Mol Biol.

[21] Peden DB, Tucker K, Murphy P (1999). Eosinophil influx to the nasal airway after local, low-level LPS challenge in humans. J Allergy Clin Immunol.

[22] Neuder LE, Keener JM, Eckert RE (2009). Role of p38 MAPK in LPS induced pro-inflammatory cytokine and chemokine gene expression in equine leukocytes. Vet Immunol Immunopathol.

[23] Weersink AJ, Van Kessel KP, Torensma R (1990). Binding of rough lipopolysaccharides (LPS) to human leukocytes. Inhibition by anti-LPS monoclonal antibody. J Immunol.

[24] Hassani M, Leijte G, Bruse N (2020). Differentiation and activation of eosinophils in the human bone marrow during experimental human endotoxemia. J Leukoc Biol.

[25] Kumari A, Singh DK, Dash D, Singh R (2019). Intranasal curcumin protects against LPS-induced airway remodeling by modulating toll-like receptor-4 (TLR-4) and matrixmetalloproteinase-9 (MMP-9) expression via affecting MAP kinases in mouse model. Inflammopharmacology.

[26] Grosicki M, Wójcik T, Chlopicki S, Kieć-Kononowicz K (2016). In vitro study of histamine and histamine receptor ligands influence on the adhesion of purified human eosinophils to endothelium. Eur J Pharmacol.

[27] Heuke S, Vogler N, Meyer T (2013). Detection and discrimination of non-melanoma skin cancer by multimodal imaging. Healthcare.

[28] Schindelin J, Arganda-Carreras I, Frise E (2012). Fiji: an open-source platform for biological image analysis. Nat Methods.

[29] Rueden CT, Schindelin J, Hiner MC (2017). Image J2: ImageJ for the next generation of scientific image data. BMC Bioinformatics.

[30] Rodewald M, Bae H, Huschke S (2021). In vivo coherent anti-Stokes Raman scattering microscopy reveals vitamin A distribution in the liver. J Biophotonics.

[31] Meijering E. FeatureJ: An ImageJ plugin suite for image feature extraction. https://imagescience.org/meijering/software/featurej/

[32] Arganda-Carreras I, Kaynig V, Rueden C (2017). Ticrainable Weka Segmentation: a machine learning tool for microscopy pixel classification. Bioinformatics.

[33] Giembycz MA, Lindsay MA (1999). Pharmacology of the eosinophil. Pharmacol Rev.

[34] Ishihara K, Takahashi A, Kaneko M (2007). Differentiation of eosinophilic leukemia EoL-1 cells into eosinophils induced by histone deacetylase inhibitors. Life Sci.

[35] Uenoyama Y, Ohshima Y, Morita M (1991). Dibutyryl cyclic AMP induces formyl peptide receptor expression and chemotactic responses in a human eosinophilic cell line, EoL-1. Exp Hematol.

[36] Nissar AU, Sharma L, Mudasir MA (2017). Chemical chaperone 4-phenyl butyric acid (4-PBA) reduces hepatocellular lipid accumulation and lipotoxicity through induction of autophagy. J Lipid Res.

[37] Colitti M, Boschi F, Montanari T (2018). Dynamic of lipid droplets and gene expression in response to β-aminoisobutyric acid treatment on 3T3-L1 cells. Eur J Histochem.

[38] Tylichová Z, Slavík J, Ciganek M (2018). Butyrate and docosahexaenoic acid interact in alterations of specific lipid classes in differentiating colon cancer cells. J Cell Biochem.

[39] Czamara K, Majzner K, Pacia MZ (2014). Raman spectroscopy of lipids: a review&nbsp;. J Raman Spectrosc.

[40] Atkins CG, Buckley K, Blades MW, Turner RFB (2017). Raman spectroscopy of blood and blood components. Appl Spectrosc.

[41] Short KW, Carpenter S, Freyer JP, Mourant JR (2005). Raman spectroscopy detects biochemical changes due to proliferation in mammalian cell cultures. Biophys J.

[42] Szafraniec E, Kus E, Wislocka A (2019). Raman spectroscopy–based insight into lipid droplets presence and contents in liver sinusoidal endothelial cells and hepatocytes. J Biophotonics.

[43] Wood BR, Caspers P, Puppels GJ (2007). Resonance Raman spectroscopy of red blood cells using near-infrared laser excitation. Anal Bioanal Chem.

[44] Taylor P, Movasaghi Z, Rehman S, Rehman IU (2007). Raman spectroscopy of biological tissues raman spectroscopy of biological tissues. Appl Spectrosc Rev.

[45] Czamara K, Majzner K, Selmi A (2017). Unsaturated lipid bodies as a hallmark of inflammation studied by Raman 2D and 3D microscopy. Sci Rep.

