# Supplementary material for: Identification of inflammatory markers in eosinophilic cells of the immune system: fluorescence, Raman and CARS imaging can recognize markers but differently
Source: Cell Mol Life Sci. 2021 Dec 22;79(1):52. doi: 10.1007/s00018-021-04058-4 (PMC8739296; doi:10.1007/s00018-021-04058-4)
Supplement: Supplementary file 1 — Supplementary file1 (DOCX 1373 KB) [file 18_2021_4058_MOESM1_ESM.docx]

SUPPLEMENTARY INFORMATION

**Identification of inflammatory markers in eosinophilic cells of the immune system. Fluorescence, Raman and CARS imaging can recognize markers but differently**

Aleksandra Borek-Dorosz^1,2^, Marek Grosicki^2^, Jakub Dybas^2^, Ewelina Matuszyk^2^, Marko Rodewald^3,4^, Tobias Meyer-Zedler^3,4^, Michael Schmitt^4^, Juergen Popp^3,4^, Kamilla Malek^1*^, Malgorzata Baranska^1,2,*^

^1^Faculty of Chemistry, Jagiellonian University, Gronostajowa 2, 30-387 Krakow, Poland
^2^Jagiellonian Centre for Experimental Therapeutics (JCET), Jagiellonian University, Bobrzynskiego 14, 30-348 Krakow, Poland
^3^Institute of Physical Chemistry (IPC) and Abbe Center of Photonics (ACP), Friedrich-Schiller-University, Helmholtzweg 4, Jena, Germany
^4^Leibniz Institute of Photonic Technology e.V., Member of Leibniz Health Technologies, Albert-Einstein-Str. 9, Jena, Germany

* correspondence to:
K. Malek (kamilla.malek@uj.edu.pl)

M. Baranska (m.baranska@uj.edu.pl)

This material includes:

Table S1.
Figure S1.
Table S2.
Figure S2.
Figure S3.
Figure S4.
Figure S5.

**Table S1**. Comparison of Eos and EoL-1 features. Blue and green – nucleus, orange - LBs, red - granules with EPO ^3,7,11,28,47–51^.

| *Feature* | *Eosinophil 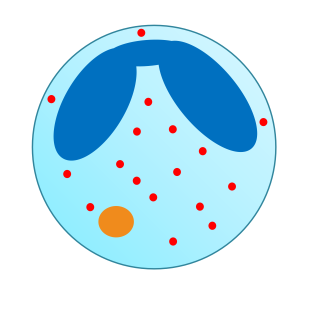* | *EoL-1 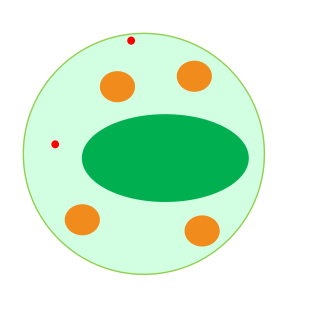* |
| --- | --- | --- |
| Nucleus | bilobed nuclei | ellipse-shaped nuclei |
| Number of lipid bodies | small portion of cells possesses single LB | high |
| Number of granules | high | 0-1 |
| Diameter of cell | ̴10-20 µm | ̴10 µm |
| Diameter of LBs | 0.5-2 µm | 0.5-4 µm |

**Table S2.** Summary of how many cells for each group of stimulated EoL-1 cells possess the given number of LBs in their cytoplasm based on CARS images analysis.

|  | BA | control | IL 1beta | LPS | TNF |
| --- | --- | --- | --- | --- | --- |
| mean | 3.89 | 1.94 | 1.82 | 1.18 | 1.51 |
| median | 3 | 1 | 1 | 1 | 1 |
| n_total, cells | 655 | 390 | 686 | 723 | 667 |
| n_LBs | **n_cells** | **n_cells** | **n_cells** | **n_cells** | **n_cells** |
| 0 | 97 | 125 | 192 | 344 | 222 |
| 1 | 83 | 77 | 172 | 160 | 177 |
| 2 | 78 | 75 | 126 | 96 | 109 |
| 3 | 87 | 44 | 80 | 64 | 87 |
| 4 | 86 | 30 | 52 | 22 | 40 |
| 5 | 59 | 10 | 30 | 18 | 16 |
| 6 | 45 | 8 | 19 | 8 | 7 |
| 7 | 28 | 10 | 10 | 7 | 5 |
| 8 | 22 | 8 | 4 | 1 | 2 |
| 9 | 23 | 0 | 1 | 2 | 2 |
| 10 | 15 | 0 | 0 | 0 | 0 |
| 11 | 6 | 0 | 0 | 1 | 0 |
| 12 | 12 | 2 | 0 | 0 | 0 |
| 13 | 7 | 0 | 0 | 0 | 0 |
| 14 | 4 | 0 | 0 | 0 | 0 |
| 15 | 1 | 0 | 0 | 0 | 0 |
| 16 | 1 | 0 | 0 | 0 | 0 |
| 17 | 0 | 0 | 0 | 0 | 0 |
| 18 | 0 | 0 | 0 | 0 | 0 |
| 19 | 0 | 0 | 0 | 0 | 0 |
| 20 | 0 | 0 | 0 | 0 | 0 |
| 21 | 0 | 0 | 0 | 0 | 0 |
| 22 | 1 | 1 | 0 | 0 | 0 |

**

**

**Figure S1.** Effect of different concentrations of butyric acid on viability of EoL-1 cells


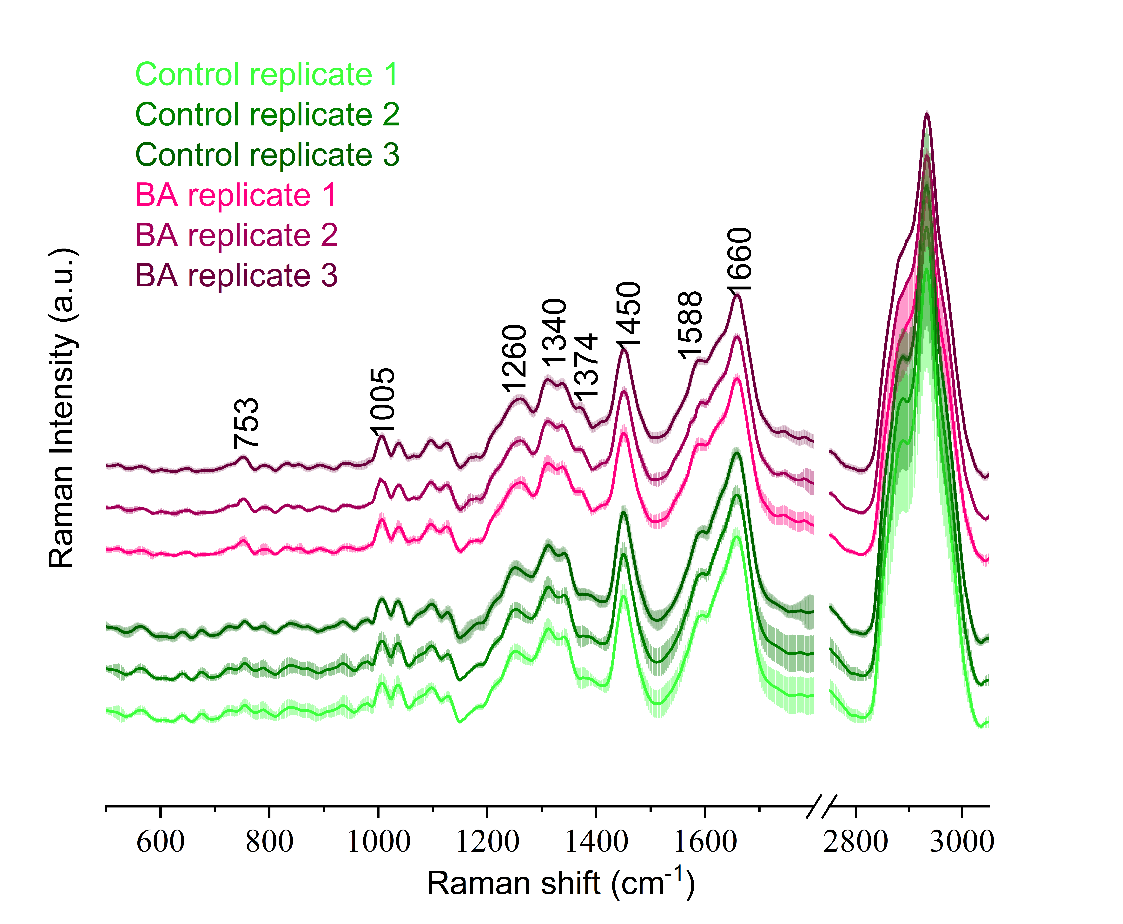


**Figure S2.** Mean spectra of the cytoplasm class for 3 replicates of control and BA cells with standard deviation (shading)


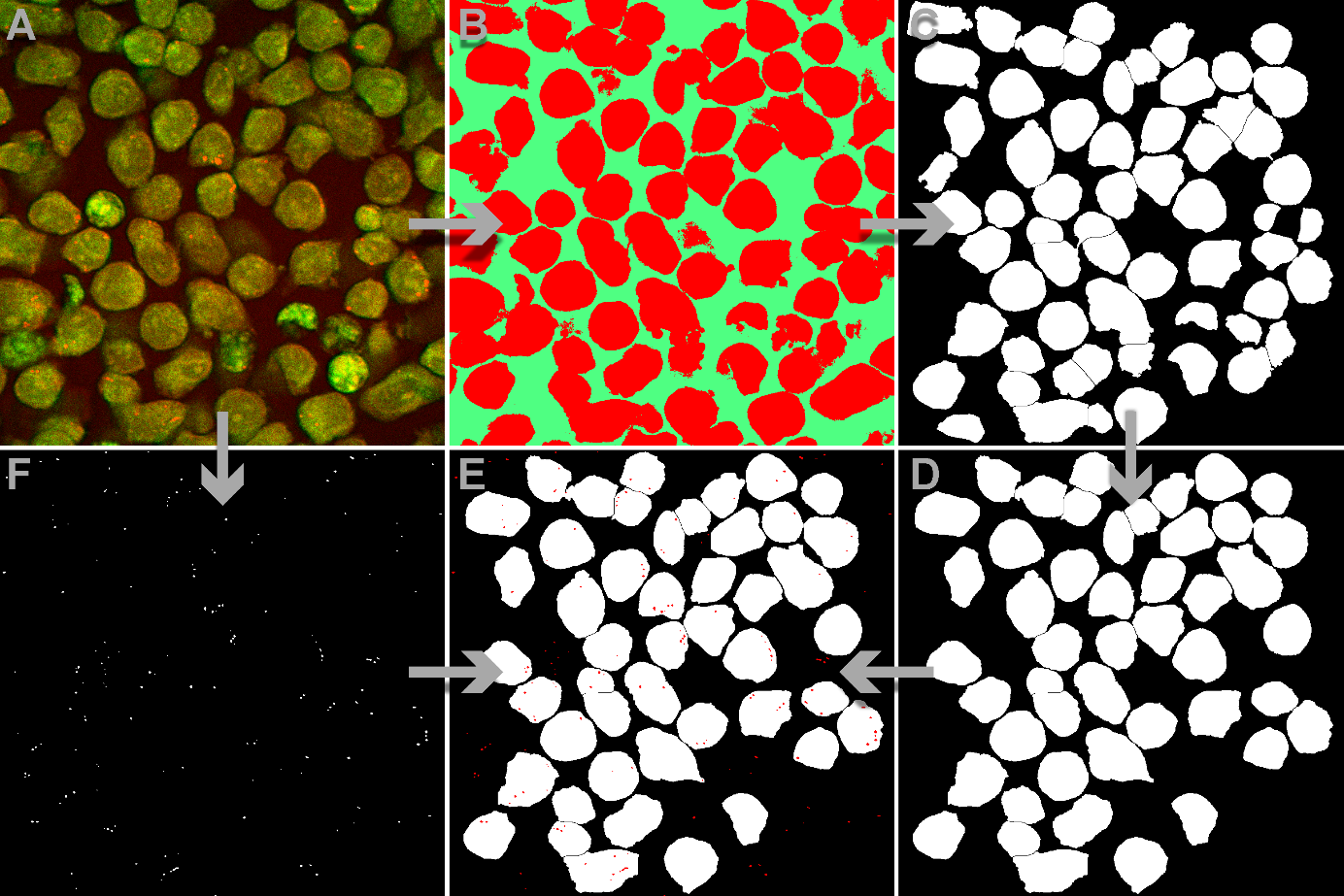


**Figure S3.** Illustration of key steps of the cell identification in CARS/TPEF images. A) red: CARS, green: TPEF; B) result of WEKA segmentation; C) result of automatic mask cleaning steps; D) result of manual mask correction, E) an overlay of final mask and LBs (F), identified in the separate procedure)


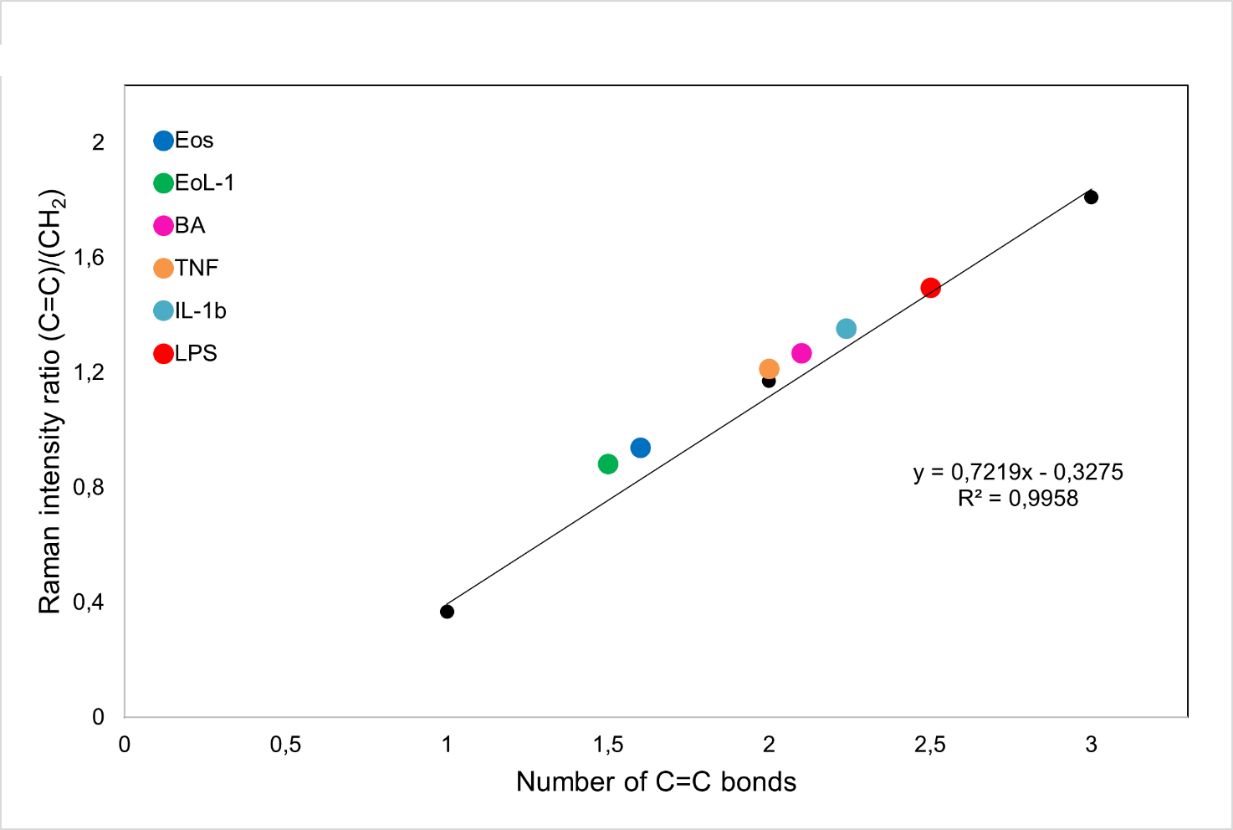


**Figure S4.** Relationship between the average number of the C=C double bonds and the intensity ratio of Raman bands at 1655 (ν(C=C)) and 1444 cm^-1^ (δ(CH_2_)) for selected unsaturated fatty acids; OA – oleic acid (18:1), LA – linoleic acid (18:2) and ALA – α-linolenic acid (18:3), the standards are marked as black spots

**
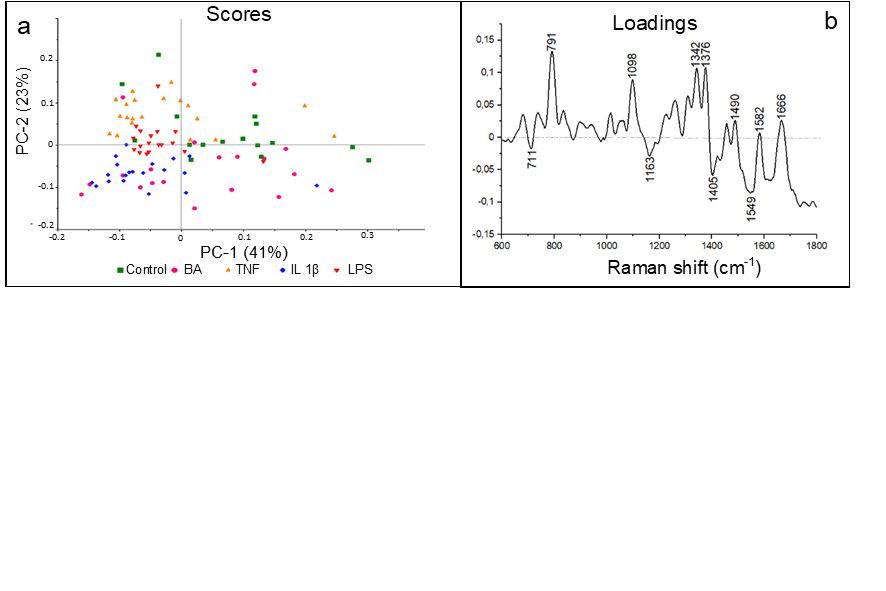
**

**Figure S5.** Scores (a) and loading plots (b) of principal component analysis of nuclei classes of stimulated and control EoL-1 cells performed in the region of 600–1800 cm^-1^
